# In Vitro Bacterial Growth on Titanium Surfaces Treated with Nanosized Hydroxyapatite

**DOI:** 10.3390/jfb16020066

**Published:** 2025-02-16

**Authors:** Maria Holmström, Sonia Esko, Karin Danielsson, Per Kjellin

**Affiliations:** 1 Promimic AB, Entreprenörsstråket 10, 431 53 Mölndal, Swedenper.kjellin@promimic.com (P.K.); 2 Department of Applied Chemistry, Chalmers University of Technology, 412 96 Göteborg, Sweden

**Keywords:** nanosized hydroxyapatite, implant, surface coating, antibacterial, biofilm, *S. epidermidis*, *P. aeruginosa*

## Abstract

Bacterial growth on implant surfaces poses a significant obstacle to the long-term success of dental and orthopedic implants. There is a need for implants that promote osseointegration while at the same time decreasing or preventing bacterial growth. In this study, the existing methods for the measurement of bacterial biofilms were adapted so that they were suitable for measuring the bacterial growth on implant surfaces. Two different strains of bacteria, *Pseudomonas aeruginosa* and *Staphylococcus epidermidis,* were used, and the in vitro effect of bacterial growth on titanium surfaces coated with an ultrathin (20–40 nm thick) layer of nanosized hydroxyapatite (nHA) was investigated. After 2 h of biofilm growth, there was a 33% reduction in both *S. epidermidis* and *P. aeruginosa* bacteria on nHA compared to Ti. For a more mature 24 h biofilm, there was a 46% reduction in *S. epidermidis* and a 43% reduction in *P. aeruginosa* on nHA compared to Ti. This shows that coating nHA onto implants could be of benefit in reducing implant-related infections.

## 1. Introduction

The use of dental and orthopedic implants is steadily increasing. A highly undesirable phenomenon in implant surgery is infection at the implantation site. Surgical site infections are troublesome, with increased patient morbidity and mortality, longer hospital stays and increased healthcare costs. The treatment is even more demanding in relation to implants and can, in the worst case, lead to implant rejection. Infections may be classified as either early-onset or late-onset, but the border between these phases varies in the literature, with anywhere from 30 days to 2 years post-surgery being classified as a late infection [1,2,3,4,5,6,7].

Bacterial strains that are known to cause infections in orthopedic implant surgery include coagulase-negative staphylococci such as *S. epidermidis*, *S. aureus*, streptococci, *P. aeruginosa*, *E. coli*, enterococci, *Cutibacterium* species and *Enterobacter* species [4,5,6,8,9]. Early infections often arise due to direct inoculation during surgery, whereas late-onset infections occur via hematogenous spread [6,8]. Generally, early infections are caused by more virulent bacterial strains, whereas late infections are due to less virulent strains [7,8].

The risk of human staphylococcal infection has been shown to be more than 10,000-fold increased in the presence of a foreign body [10], and in a guinea pig infection model, an inoculum of *S. aureus* of as little as 100 Colony Forming Units (CFUs) in the presence of subcutaneous implants was found to cause an infection, whereas 10^8^ CFUs could be cleared by the immune system without signs of infection in the absence of a foreign body [11]. A lowered bacterial number causing infections in the presence of implants has also been observed in other animal infection models, as well as in patients [12,13,14,15]. This may be due to several factors, in addition to the immune response and frustrated phagocytosis associated with implants. Biofilm bacteria can alter the immune response to evade elimination using quorum-sensing molecules [16]. Staphylococcal biofilms have been shown to drive the immune response into an anti-inflammatory and fibrotic response and alter both neutrophil and macrophage function [16,17,18,19]. The immune response also alters depending on the implant topography and the bacterial species present [20].

Infection rates depend on patient factors, surgery procedures and hygiene routines [21], as well as the implant design [22]. Antibiotic treatment, both systemic and local, is one strategy for reducing infection rates. Another approach is to modify the implant’s surface. One can distinguish between two different types of methods for combatting bacterial growth on an implant surface: bacteriostatic and bactericidal. Both approaches are commonly used together. In the bacteriostatic approach, the surface prevents the growth of bacteria. This can be achieved by creating superhydrophilicity [23] through surface topographical changes and by applying antifouling coatings, such as polymer brushes [24]. A smoother surface lowers the surface area and thereby decreases the amount of attachment points for the bacteria [25]. This method is used for external fixation pins [26] and dental abutments [22]. However, in patients, many factors influence the risk of implant infections, and significant differences between smooth, moderately rough and rough surfaces when it comes to biofilm formation and peri-implantitis are not always observed [27,28].

The bactericidal approach involves modifying the implant’s surface to kill bacteria. Noble metal coatings [29,30], coatings containing antibiotics [31] and hydrogels [32] have been successfully used to induce a bactericidal effect, as has the use of quaternary ammonium compounds [33] and nanopatterning of surfaces, such as creating nano-pillars and nano-pores [34].

Apart from infection, insufficient bone-to-implant integration is another factor that increases the failure rate of dental and orthopedic implants, and to improve the integration, the properties of the implant surface are of vital importance. Factors such as micro-roughness, nano-roughness, chemical composition and physical and mechanical factors all affect osseointegration [35]. However, the factors that are known to affect osseointegration are often similar to the properties that affect bacterial growth, especially regarding surface texturing techniques. Making an implant surface rougher through blasting and/or acid etching is known to promote osseointegration, but it also provides a great substrate for bacterial growth. Conversely, a polished smooth surface that displays a low bacterial growth rate often has poor osseointegration. Ideally, an implant surface should display fast osseointegration and also have antibacterial properties, and the challenge is naturally to combine these properties.

Hydroxyapatite (HA) coatings are well known to improve implant osseointegration [36,37]. Thick (>50 µm) plasma-sprayed HA was introduced on orthopedic implants in the 1980s, with a significant effect on implant integration. In later years, some concerns were raised about the clinical problems with thick HA coatings, such as delamination and wear [38,39], and it was suggested that thinner coatings may be better in stimulating osseointegration without these risks. Implant treatment using ultrathin coatings of nHA is known to have a significant effect on osseointegration [40,41,42], even in compromised bone [43,44]. However, the effect of bacterial growth on nHA is not well studied.

There are many different ways to study bacterial biofilm growth, including static and flow cell models, culturing and microscopy studies. For the sake of simplicity, surface-coated discs are often coated on one side and placed in well plates or flasks for biofilm growth; then, biofilm removal is performed using ultrasound treatment. However, for this procedure, the question arises of what is growing on the non-coated part of the disc. This may pose a problem, and coating both sides of the discs is not always a viable option.

The aim of the current study was, therefore, to refine the existing methods to investigate bacterial growth on surfaces and to use this method to investigate the bacterial growth on Ti substrates, with or without a coating of ultrathin (20–40 nm) nHA. The null hypothesis was that there was no difference between Ti and nHA-coated Ti. For this purpose, a drop method was used, placing a liquid drop containing bacteria onto discs for growth. In addition, a well plate was created to press onto the Ti and nHA-modified Ti disc, only to expose the top surfaces to bacteria. A bottom plate was placed below the Ti discs, and everything was held together by screws. A bacterial count of 1× 10^6^ or 50,000 CFU/mL of *S. epidermidis* or *P. aeruginosa* was used and allowed to form 2 or 24 h biofilms before biofilm removal and culturing.

## 2. Materials and Methods

### 2.1. Surface Preparation

Two types of Ti discs were used in this study: 1 cm Ø discs of Ti grade 4 and 2 cm Ø discs of Ti grade 2 (Kullbergs Mikroteknik AB, Lycke, Sweden). The larger diameter was chosen to improve the statistics for the 24 h experiments; Ti grade 2 and grade 4 both consist of Ti, with no difference in biological response, but grade 4 has a higher mechanical strength. Both grades are frequently used for implants. All discs were ground using P400 grit sandpaper (Biltema, Partille, Sweden), ultrasonically cleaned for 10 min per step using isopropanol (Fisher Scientific, Hampton, NH, USA, 99%), 1 M HNO_3_ (Scharlau, Barcelona, Spain, 65%), H_2_O type 1 and isopropanol. The 2 cm discs were also thermally cleaned for 5 min at 450 °C (Nabertherm, Lilienthal, Germany) to obtain a more homogenous nHA layer. This procedure was performed for both the control discs and the discs to be coated with nHA. Then, the discs were either used as Ti controls or surface treated with nHA. In short, the nHA layer was applied through spin coating; an in-house synthesized coating liquid containing nHA (40–80 µL) was applied to the surfaces, which were then placed on a spin coater using a spin speed of 2500 rpm for 3 s and finally thermally treated at 450 °C. A more thorough description of the coating procedure and coating liquid is provided in reference [42].

### 2.2. Surface Characterization

Scanning electron microscopy (SEM) and energy dispersive X-ray analysis (EDX) were performed using a Zeiss FEG-SEM Sigma (Oberkochen, Germany) with Gemini optics and equipped with EDX. Three discs were studied, and 3 EDX spectra were obtained for each disc, giving a total of 9 spectra for each type of disc.

As presented below, 24 h bacterial biofilms were also studied using SEM. Before SEM analysis, the biofilms were left overnight in 4% buffered paraformaldehyde (VWR, Radnor, PA, USA) and then rinsed 3 times in phosphate-buffered saline (PBS) (Sigma Aldrich, St. Louis, MO, USA, 0.01 M) before being subjected to a drying gradient of 50, 60, 70, 80, 90 and 100% ethanol (Fisher Scientific, Hampton, NH, USA, ≥99%) for 10 min per step. The samples were then left in 50% hexamethyldisilazane (Thermo Fisher, Waltham, MA, USA, 98%) in ethanol for 20 min and finally left in 98% hexamethyldisilazane and allowed to dry in ambient air. Before SEM analysis, the samples were gold-sputtered for 60 s, 10 mA (Emitech K550X, Quorum, Laughton, UK), yielding a gold layer of 3 nm according to the manufacturer.

To investigate the nHA layer thickness, an extremely smooth titanium-deposited (PVD method) silicon wafer was coated with the nHA surface and studied in a transmission electron microscope (TEM, FEI Tecnai T20, FEI, Hillsboro, OR, USA) in bright field mode at 200 kV acceleration voltage. For the nHA sample to be analyzed using TEM, an electron-transparent thin foil from the sample was prepared using a combined focused ion beam and scanning electron microscope workstation (FEI Versa 3D). Over the region of interest, a 3 × 12 µm Platinum layer of 2 µm thickness was deposited first by using electron beam deposition (2 kV) and then ion (Ga^+^) beam deposition (50 pA, 30 kV). Platinum deposition occurred by scanning the electron or ion beam over the area of interest so that secondary electrons interacted with and decomposed an organometallic precursor gas that was injected into the chamber. Two trenches were subsequently milled out (5 nA, 30 kV) on both sides of the Pt layer. The sample was then tilted, and the foil was milled loose on three sides (3 nA, 30 kV). A sharp (radius < 2 µm) tungsten micromanipulator needle (Omniprobe, Oxford Instruments, Abingdon, UK) was inserted into the chamber and attached to the sample foil. The remaining side connecting the foil with the substrate was milled off. The foil was then attached to a copper TEM half-grid by means of Platinum deposition. The foil was then thinned to a thickness of less than 100 nm using decreasing ion currents and acceleration voltages (from 0.5 nA, 30 kV down to 7.7 pA, 5 kV).

To evaluate the surface roughness, white light coherence scanning interferometry was performed according to ISO 25178-604 [45] using a MicroXAM (ADE Phase Shift Technology, Tuscon, AZ, USA) with a magnification of 50×. Two discs from each substrate were used, and for each disc, three areas were measured.

Static contact angle measurements were performed to investigate the wettability of the samples. First, 5 µL drops of type 1 water [46] (Elga LabWater, High Wycombe, UK, 18.2 MΩ-cm) were dropped onto the surfaces, either the Ti controls or nHA-coated Ti. Photographs were obtained, and contact angles were determined manually using software (ImageJ, version 1.54 g Java 1.8.0_345 (64 bit)). Three drops were tested for each substrate, with one drop per disc.

### 2.3. X-Ray Diffraction Analysis

A powder produced directly from the coating liquid was analyzed using powder XRD. A Bruker D8 Discover was used (Billerica, MA, USA). The XRD analysis was performed using Cu-K_α_ radiation (λ = 1.5418 Å) with a 2θ between 20° and 60° (step size 0.020°) for a total analysis time of 28 min. As a hydroxyapatite reference pattern, COD 96-900-2214 of the Crystallographic Open Database was used.

### 2.4. Calcium Assay

A colorimetric calcium assay was performed to investigate the amount of calcium ions on the nHA-coated surfaces, which, in turn, can be used to calculate the approximate thickness of the nHA coating. First, 12 mm nHA-coated discs were dipped in 5 mL 0.01 M HNO_3_ (Scharlau, Barcelona, Spain, 65%) for 5 min; this procedure dissolved all nHA that was present on the disc. Then, 5 mL of a solution consisting of 60 µM ArsenazoIII (Sigma Aldrich, St. Louis, MO, USA) in 0.1 M Tris solution (Fisher Scientific, Hampton, NH, USA) was added; this step increased the pH of the solution. The calcium–dye complex turned purple-red, and its absorbance was immediately measured at 650 nm (Jenway 7315 Spectrophotometer, Cole-Parmer, Vernon Hills, IL, USA) and compared to a standard curve.

### 2.5. Bacterial Strains and Culture

*S. epidermidis* CCUG 39,508 and *P. aeruginosa* CCUG 56,489 were grown on Brain heart infusion (BHI) agar (Fisher Scientific, Hampton, NH, USA) at 37 °C and then stored in a fridge at 4 °C. These species were chosen as they are common Gram-positive and Gram-negative species causing implant-associated infections. Colonies were then transferred to BHI medium, cultured overnight at 37 °C to stationary phase, and used for 2 h biofilm experiments. For 24 h biofilms, overnight samples were immediately used if in mid-logarithmic phase; otherwise, they were transferred to fresh BHI and re-cultured into mid-exponential growth phase, corresponding to Abs_600nm_ between 0.5 and 0.7 (Jenway 7315 Spectrophotometer). The time points 2 and 24 h were chosen in order to obtain one early and one mature biofilm.

### 2.6. Biofilm Assay

The samples for 2 h biofilms were diluted to 1 ×10^6^ bacteria/mL in BHI, and 80 µL (80,000 bacteria) was added as a drop onto the top of 1 cm Ø discs and cultured for 2 h at 37 °C in air before biofilm removal. The drop method enabled biofilm growth on only the coated surface, but it had limitations on how long culturing was possible due to drying effects.

**Figure 1:**
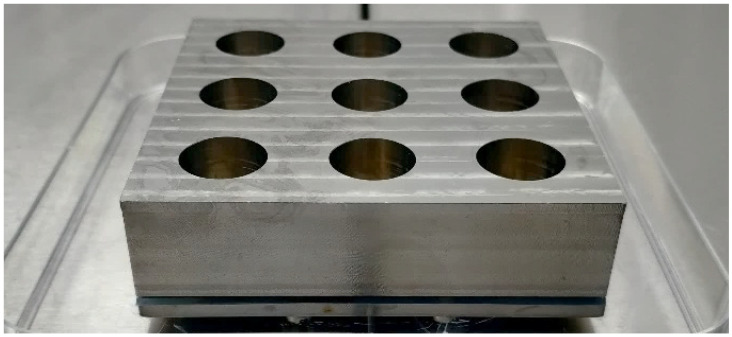
Assembled 9-well plate.

For the 24 h biofilms, 9-well metal plates were created to culture biofilms on one side of the discs only. The well plates consisted of a top part with holes to create the wells, into which 2 cm Ti discs were fitted with a silicone O-ring. Below the discs, a silicone sheet was fitted to maintain the liquid inside the wells, supported by a Teflon plate and a solid metal bottom plate, all of which were held together by screws; see Figure 1. For these tests, 2 cm discs were used instead of 1 cm to increase biofilm surface areas and statistical accuracy.

Log phase bacteria for 24 h studies were ultrasonically homogenized for 10 s at 20% power (Bandelin Sonopuls, Probe MS72, Bandelin electronic GmBH & Co., Berlin, Germany) and diluted to 50,000 bacteria/mL in BHI using a Bürker Türk counting chamber (Brand GmbH, Wertheim, Germany) and an optical microscope (Zeiss Axioskop 40, Oberkochen, Germany). A total of 1 mL bacterial solution was added to each disc and cultured for 24 h at 37 °C in air.

After growth, the discs were rinsed in 1 mL PBS (0.01 M, Sigma Aldrich, St. Louis, MO, USA). The 1 cm discs were transferred to 1 mL fresh PBS, and the 2 cm discs were transferred to 2 mL PBS. For 24 h *P. aeruginosa*, the discs were pipetted 5 times to remove visible biofilm from the discs. Then, all the discs were ultrasonicated for 5 min (Elmasonic S 80 H, 37 kHz, Elma Schmidbauer GmbH, Singen, Germany) for biofilm removal. Due to the presence of visible bacterial aggregates, 24 h *P. aeruginosa* solutions were then ultrasonically homogenized for 10 s at 20% power (Bandelin Sonopuls, Probe MS72, Bandelin electronic GmBH & Co., Berlin, Germany) to dislodge the agglomerates. The bacterial solutions were then serially diluted in PBS, and 7 × 10 µL drops per dilution were cultured on BHI agar plates at 37 °C in air overnight, according to the drop plate method. Seventeen different 2 h triplicate biofilm experiments were performed, yielding an *n*-value of 51. For the 24 h biofilms, 8 experiments were performed in triplicates or quadruplicates, yielding an *n*-value of 30.

### 2.7. Confocal Microscopy

The confocal microscope used was a ZEISS LSM 980 with Airyscan 2 (Oberkochen, Germany). Samples were stained using LIVE/DEAD *Bac*Light, kit L13152 (Invitrogen, Waltham, MA, USA), with propidium iodide and Syto 9, prepared according to the manufacturer. A total of 10 µL was added to each disc and incubated in the dark at room temperature for 15 min before observation.

### 2.8. Statistical Analysis

A two-sided student’s *t*-test for unpaired samples of unequal variance was utilized for statistical analysis. Any *p*-value below 0.05 was considered statistically significant.

## 3. Results

### 3.1. Surface Characterization

**Figure 2:**
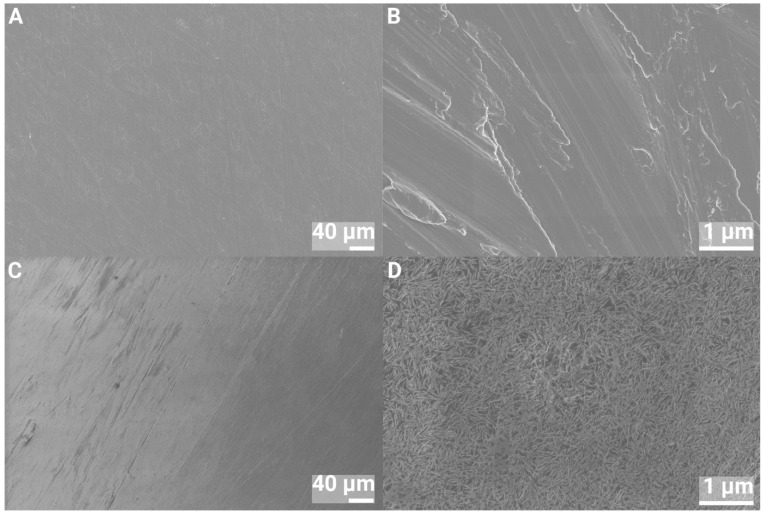
SEM images of 1 cm Ti discs at 500× (**left**) and 40,000× (**right**) magnification. (**A**,**B**) Ti and (**C**,**D**) nHA-coated Ti.

**Figure 3:**
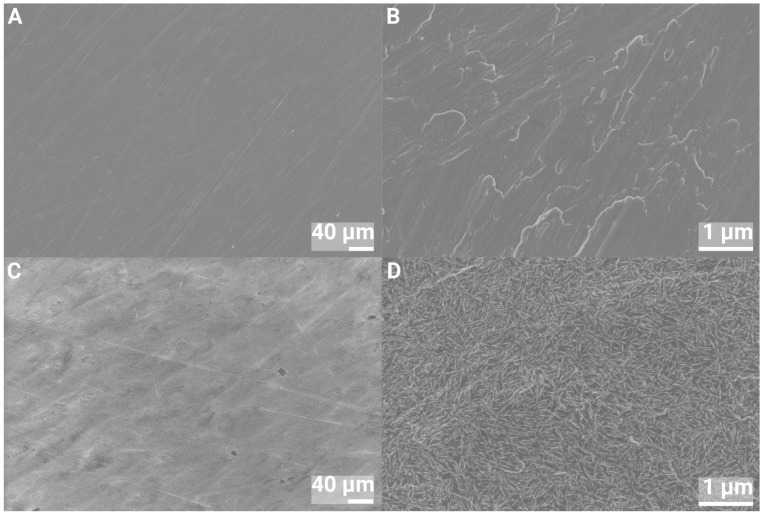
SEM images of 2 cm Ti discs at 500× (**left**) and 40,000× (**right**) magnification. (**A**,**B**) Ti and (**C**,**D**) nHA-coated Ti.

SEM images of the 1 and 2 cm discs of pure Ti and nHA-coated Ti are shown in Figure 2 and Figure 3. At 500× magnification, both surfaces looked smooth, whereas at 40,000× magnification, the nHA-treated Ti was shown to be covered by a homogeneous layer of HA crystals. The average crystal size was estimated from SEM imaging to be 100 ± 20 nm in length and 6 ± 2 nm wide.

**Figure 4:**
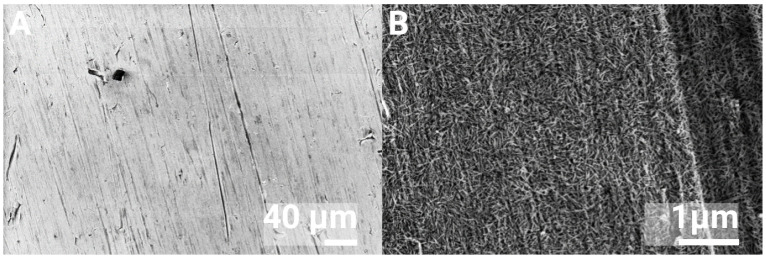
SEM images of the nHA coating on discs used for the Ca assay, at 500× (**A**) and 40,000× (**B**) magnification.

This crystal size was in a similar range as previous studies [42,47]. A 12 mm nHA-coated disc used for the calcium assay is seen in Figure 4, which shows the same nHA crystals and similar coverage as the other discs.

The EDX data in Table 1 show that the elemental composition of nHA-treated Ti grade 4 contains 0.42% calcium and 0.32% phosphorus, in addition to titanium, oxygen and carbon, whereas the grade 2 discs contained 0.25% calcium and 0.19% phosphorus. An increase in oxygen content for the heat-treated discs was also observed; this effect is most likely due to an increase in the TiO_2_ layer thickness.

**Figure 5:**
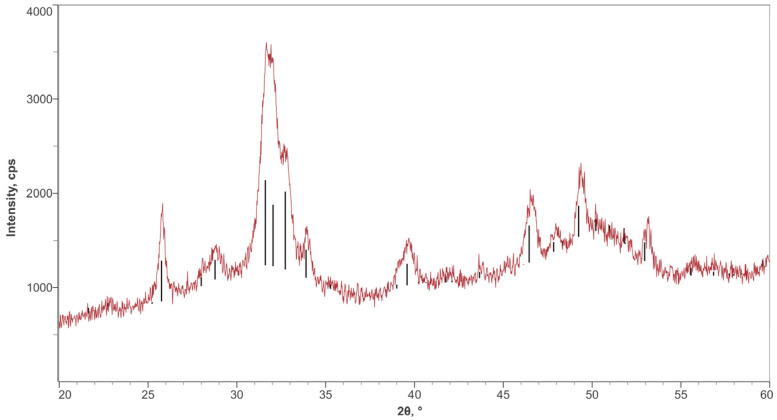
XRD diffractogram confirming HA in the nHA coating. Red represents the tested nHA, and black represents the reference peaks for HA.

To confirm the presence of hydroxyapatite, an XRD analysis was performed, and the resulting diffractogram was identified as hydroxyapatite (COD 96-900-2214). The broad peaks in the diffractogram were a result of the small crystal size, as shown in Figure 5.

The calcium assay was performed on eight discs, which gave an average calcium ion content of 3.2 µg/cm^2^. With an HA molecular weight of 502.31 g/mol, this corresponds to 8 µg HA/cm^2^. Assuming a solid layer and a density for HA of 3.15 g/cm^3^, a rough estimate of the layer thickness can be calculated. An HA content of 8 µg/cm^2^ then yields a thickness of 25 nm.

**Figure 6:**
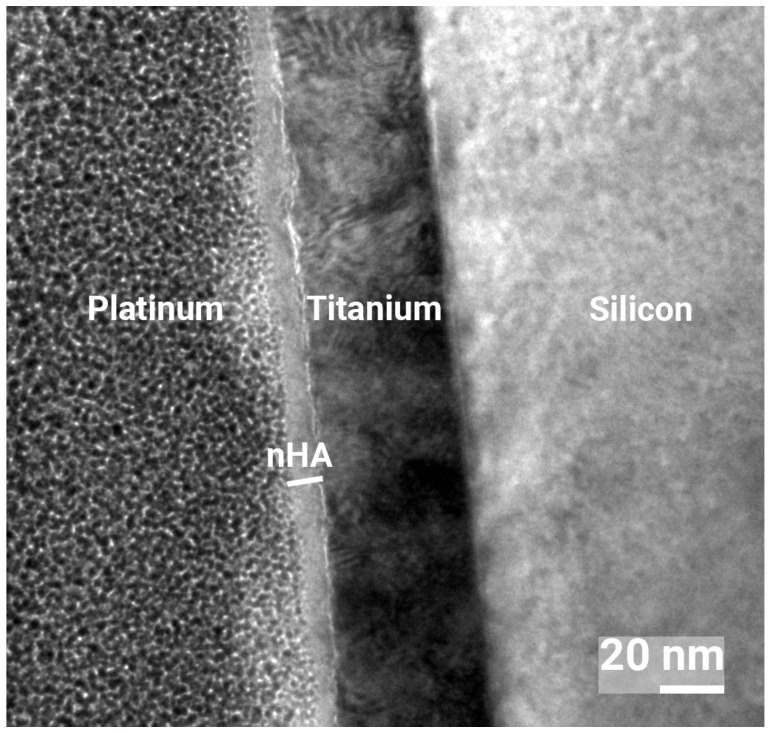
TEM cross-sectional image of nHA coating at 800,000× magnification showing a 10–20 nm thick nHA layer.

The Ca assay-calculated value was further investigated using TEM analysis of a cross-section of the nHA layer. From this analysis, the nHA layer was determined to be 10–20 nm, as shown in Figure 6.

The values of the surface roughness measured using interferometry can be found in Table 2. All surfaces had Sa values of 0.21–0.27, which are considered as smooth implant surfaces, but Ti grade 2 was somewhat rougher than Ti grade 4 [48]. Sdr values for Ti grade 2 were slightly higher than for Ti grade 4, indicating it contained finer features. The summit density (Sds) was low for all surfaces, but nHA had marginally higher Sds for both titanium grades, indicating the presence of somewhat more peaks per surface area.

**Figure 7:**
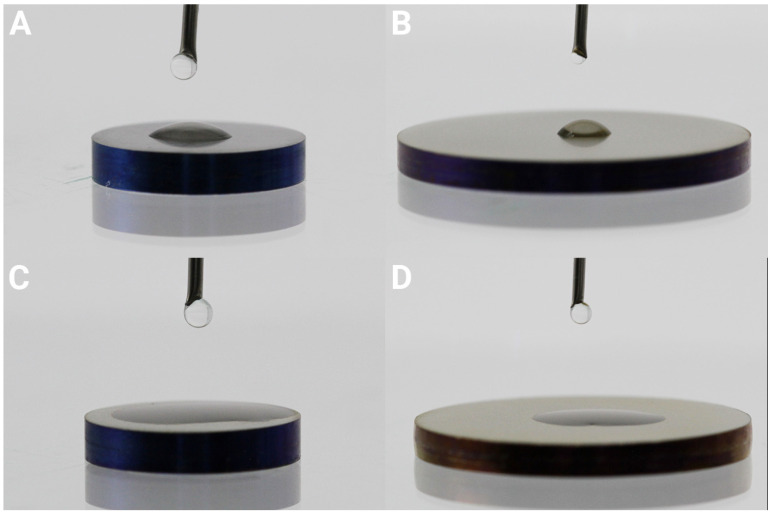
Water contact angle of 1 cm Ø (**A**,**C**) and 2 cm Ø (**B**,**D**) discs, control Ti (**A**,**B**) and nHA-coated Ti (**C**,**D**) showing the superhydrophilicity of the nHA coating.

The contact angle measurements showed the nHA-coated Ti to be superhydrophilic (contact angle < 10°), whereas the Ti controls were hydrophilic with a contact angle of 39 ± 0.5° for the 1 cm Ti discs (grade 4) and 53 ± 5.5° for the 2 cm Ti discs (grade 2); see Figure 7.

### 3.2. Confocal Laser Scanning Microscopy

**Figure 8:**
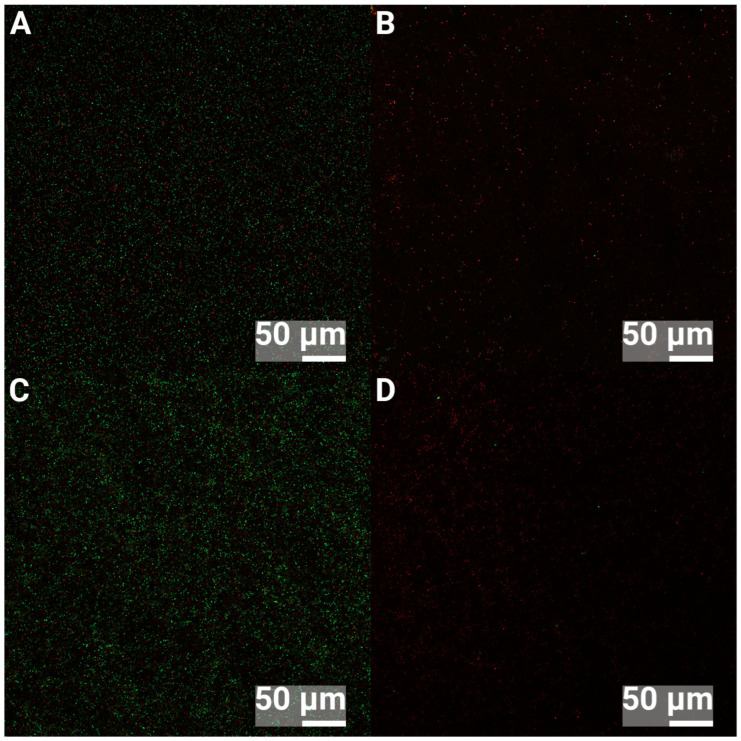
CLSM images of 24 h biofilms of *S. epidermidis*. (**A**) shows Ti and no ultrasound treatment, (**B**) shows Ti and 5 min ultrasound treatment, (**C**) shows nHA and no ultrasound treatment and (**D**) shows nHA with 5 min ultrasound treatment. Green bacteria are alive, and red bacteria are dead.

**Figure 9:**
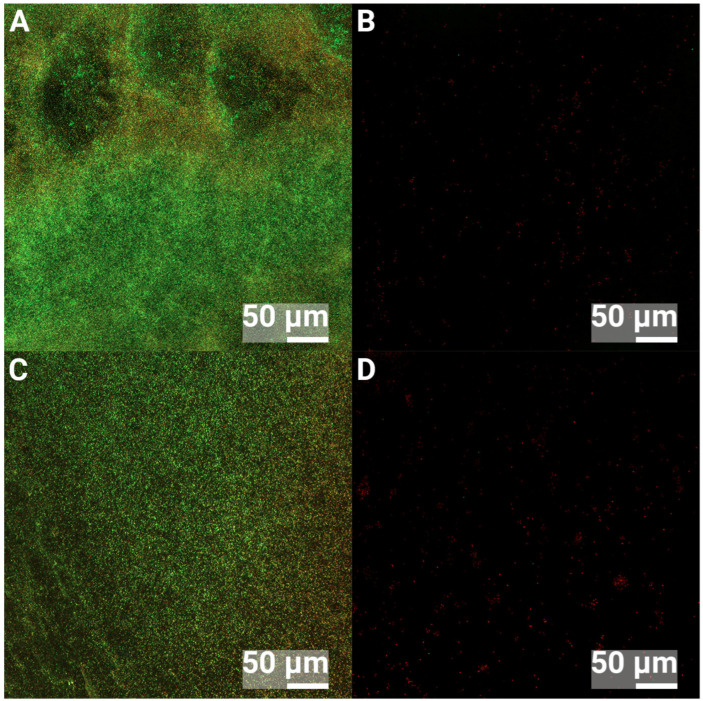
CLSM images of 24 h biofilms of *P. aeruginosa*. (**A**) shows Ti and no ultrasound treatment, (**B**) shows Ti and 5 min ultrasound treatment, (**C**) shows nHA and no ultrasound treatment and (**D**) shows nHA with 5 min ultrasound treatment. Green bacteria are alive, and red bacteria are dead.

Ultrasonic removal of bacteria from surfaces has shown superior results for biofilm removal [49,50]; however, one concern in the present study was that bacterial removal was insufficient, which would lead to measurement errors. If bacteria stick harder to the nHA-treated surfaces compared to the Ti surfaces, fewer bacteria would then be cultured, and hence, an erroneous, lower number of bacteria would be the result. Therefore, Confocal Laser Scanning microscopy (CLSM) was performed to investigate the efficiency of the ultrasonic removal of biofilms from the control Ti and nHA-coated Ti. Images were obtained before and after ultrasound treatment for 5 min at 37 kHz. The control samples that were not ultrasound-treated showed 24 h biofilms of *S. epidermidis* or *P. aeruginosa* covering the substrates, as shown in Figure 8 and Figure 9, where green bacteria were alive and red bacteria were considered dead or membrane compromised. After the ultrasound treatment, most bacteria were removed from both Ti and nHA. However, it was observed that slightly more bacteria were present on the ultrasound-treated nHA compared to Ti, but most of the retained bacteria were dead and would thus not affect the culturing results. Dead or compromised bacteria may have attached and deformed on the surface, and thus, they were stuck harder to it.

### 3.3. Biofilm Assay

To investigate the potential action of nHA, both early and more mature biofilms were studied using a biofilm assay. For the 2 h biofilms, a drop of bacteria was added to the top surface of the Ti disc. This setup was performed to prevent any misleading results from bacteria adhering to the sides and bottom of the discs that had no nHA treatment, as is the case when placing discs in well plates and adding bacteria in the well.

**Figure 10:**
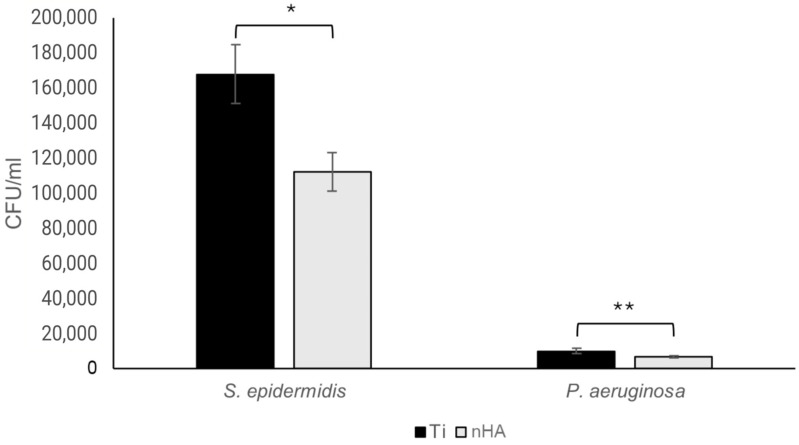
For 2 h *S. epidermidis* biofilms, there was a statistically significant 33.1% reduction (* *p* = 0.007) in bacterial growth on nHA compared to Ti, whereas there was a 33.0% reduction (** *p* = 0.033) for *P. aeruginosa*. Bars show the mean ± the standard error of the mean.

As can be seen in Figure 10, for both *S. epidermidis* and *P. aeruginosa*, there was a 33% reduction in bacteria on the nHA-treated Ti compared to pure Ti (*p* = 0.007 for *S. epidermidis* and *p* = 0.033 for *P. aeruginosa*).

**Figure 11:**
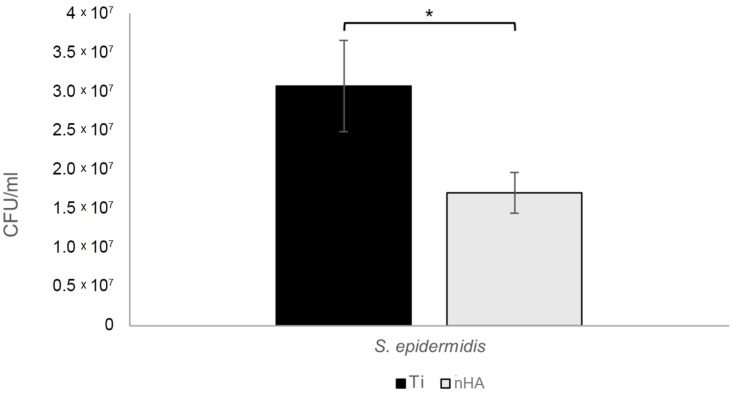
For 24 h *S. epidermidis* biofilms, there was a statistically significant 44.5% reduction (* *p* = 0.025) of bacterial growth on nHA compared to Ti. Bars show the mean ± the standard error of the mean.

**Figure 12:**
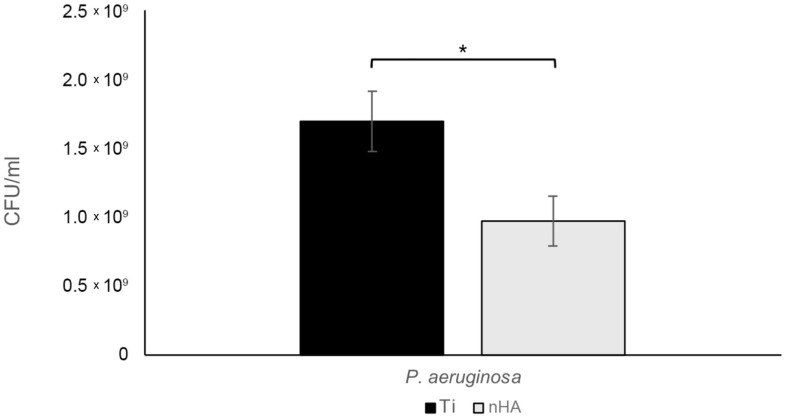
For 24 h *P. aeruginosa* biofilms, there was a statistically significant 43.0% reduction (* *p* = 0.013) in bacterial growth on nHA compared to Ti. Bars show the mean ± the standard error of the mean.

Interestingly, *S. epidermidis* grew faster during the two hours than *P. aeruginosa*, resulting in a rather large discrepancy in the final bacterial content, even though the same number of bacteria was added at the start. After 24 h, the results were reversed, with more *P. aeruginosa* than *S. epidermidis* biofilm bacteria. Studying the 24 h biofilms, there was a statistically significant 45.5% reduction in *S. epidermidis* (*p* = 0.025), as shown in Figure 11, and a 43.0% reduction in *P. aeruginosa* (*p* = 0.013), as shown in Figure 12.

### 3.4. SEM of Biofilms

**Figure 13:**
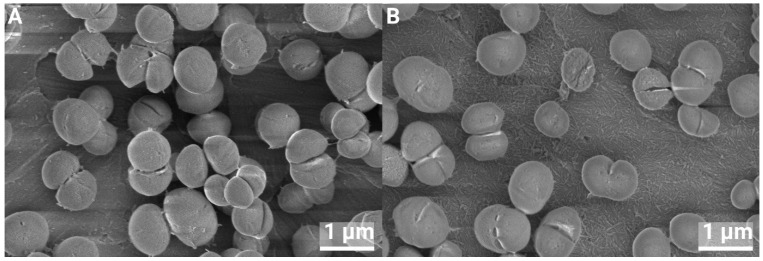
*S. epidermidis* growth on Ti (**A**) and nHA-treated Ti (**B**) for 24 h, at 40,000× magnification.

**Figure 14:**
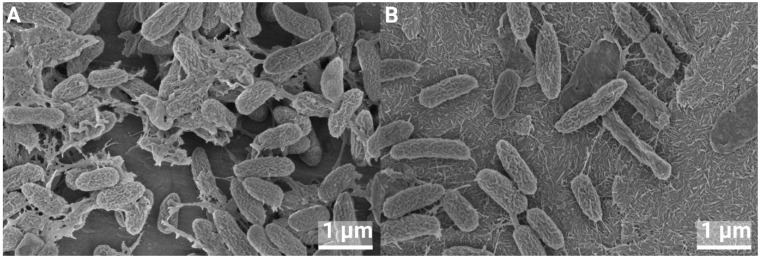
*P. aeruginosa* growth on Ti (**A**) and nHA-treated Ti (**B**) for 24 h, at 40,000× magnification.

SEM analysis was performed on 24 h biofilms. There were no visible differences in the appearance of individual bacteria on pure Ti compared to nHA-treated Ti, and cell division was observed for both *S. epidermidis* and *P. aeruginosa*; see Figure 13 and Figure 14. A few lysed bacteria were observed on the surfaces, although somewhat more lysed *P. aeruginosa* than *S. epidermidis* were found. This could be due to their thinner cell wall being more affected by the drying steps and the vacuum of the SEM. Since the bacteria, in general, did not appear compromised, the nHA seemed to be bacteriostatic rather than bactericidal.

## 4. Discussion

The surface characterization with SEM revealed nHA crystals to be homogeneously distributed all over the Ti substrate. The cross-sectional analysis performed with TEM showed an nHA layer thickness of 10–20 nm. However, as the TEM analysis was performed on an extremely smooth Si wafer, the nHA layer was expected to be thicker on rougher substrates, such as the ground Ti discs. The calculated value from the Ca assay on the discs was 25 nm, and it could be estimated to be up to 40 nm depending on substrate roughness and geometry. Two titanium grades were used in this study: Ti grade 2 and 4. Ti grade 2 was slightly rougher than Ti grade 1, and when coated with nHA, the summit density was a bit higher, indicating the presence of slightly more peaks. As Ti grade 2 is softer than grade 4, the results can be due to the grinding process using P400 paper. The nHA was superhydrophilic, which has been shown to increase interactions with biological tissue and subsequent osseointegration [51]; this would be of importance in a clinical setting.

In general, bulk HA is not regarded as an antibacterial material, but as the size of the HA is decreased to the nano-region, properties change. Studies using HA nanoparticles in the range of 19–200 nm demonstrated antibacterial effects against several bacterial species in the planktonic phase or on agar plates, such as *E. faecalis*, *E. coli*, *S. aureus*, *Bacillus* sp. and *S. mutans* [52,53,54,55]. Nanosized HA pressed into cylindrical samples and then sintered showed antibacterial effects against *S. epidermidis*, *S. aureus* and *P. aeruginosa,* depending on the sintering temperature [56]. Biofilm bacteria from pooled salivary samples showed reduced growth in the presence of nHA rods coated on Ti [57]. These studies showed a partial eradication of bacteria using nHA, with a bacterial elimination varying from 33 to 46%, depending on time and species. Interestingly, in this study the antibacterial effect was similar for both Gram-positive and Gram-negative bacteria, which is essential in a clinical setting where bacterial contamination may vary. As with all in vitro tests, the limitation is whether this effect is also visible in vivo; however, using this nHA coating on implants has the potential to reduce the number of infections related to implants.

In the literature, a vast array of different kinds of nHA is described, and some studies also report an increase in bacterial growth. Although antimicrobial against planktonic *C. albicans* after 24 h growth, an initial load of 1.5 × 10^8^ CFU/mL of *S. mutans* and *L. rhamnosus* increased in growth when HA nanoparticles were present [58]. It was hypothesized that a large bacterial load crowded the surface and created a monolayer biofilm, to which new bacteria that did not sense the surface effect could attach. It is, therefore, reasonable to believe that a high bacterial load makes it difficult to distinguish the growth of bacteria in the bulk from the growth on the surface of a substrate; therefore, the bacterial load was set to a lower amount in the present study.

For in vitro studies, this lower bacterial load is more clinically relevant, as the potential number of contaminating bacteria during implantation surgery is often low. Implant surgery should take place in ORs with ultraclean air, where there are ≤10 CFU/m^3^ [59]; 270 CFUs attached to airborne particles have been estimated to fall onto a 250 cm^2^ wound area during hip replacement [60]. Another study investigating airborne particulate contaminations during 13 hip arthroplasties showed a total CFU count of 1786, with higher numbers for longer surgery durations and higher staff counts [61]. In patients undergoing orthopedic trauma surgery, cutting the skin after disinfection and swabbing the cut yielded 4–9000 CFUs, with a median of 8 [62]; in total knee arthroplasties, the mean bacterial contamination level was 10.6 CFU/g [15]. A low initial bacterial load is also applied in many animal infection models. A rabbit spinal implant infection model using MRSA showed consistent local infection from 10^3^ CFUs and higher. In a similar setup, *E. coli* consistently produced infection using 10^5^ CFUs. In rats, 10^6^ *S. aureus* showed consistent infections in a spinal model, whereas for dogs, it was 10^2^ CFUs *S. aureus* [14].

Intraoperative contamination during implant surgery is common, and the main sources are the skin of patients and airborne particles from personnel. Even a low number of bacteria adhering early to the implant may interfere with the bone healing process. Both the antibacterial effect shown here and the ability of nHA to osseointegrate, even in metabolically challenged patients [63], improve the odds for the immune system and antibacterial therapy to eradicate potential bacteria and prevent infections.

Attempts to find out the antibacterial mechanism of hydroxyapatite have been made for nanoparticles in solution. The most common proposed mechanism is the uptake of particles by bacteria with subsequent disruption of DNA replication, formation of reactive oxygen species and direct damage to cell membranes [64]. Another possible mechanism is dissolved calcium and phosphate ions exerting an antibacterial effect [53,64], which causes damage to the cell wall and altered permeability [65,66].

The dissolution of the nHA coating may be one explanation for the observed bacteriostatic effect, but the SEM analysis after 24 h showed no effect on the appearance of the crystalline nHA layer. Even if the crystals were still visible, some dissolution on the surface of the crystals could occur. However, given that the total amount of nHA was measured to be around 8 µg/cm^2^, and TEM analysis showed the nHA layer to be 10–20 nm thick, the amount of Ca and P from the surface dissolution of the crystals would be extremely small. Therefore, a bacteriostatic effect resulting from Ca and P release is a less plausible explanation. Superhydrophilicity may be another explanation, which is well known to inhibit bacterial attachment [67], although a complete eradication of bacteria is not always obtained [23]. Yet another factor is the surface charge. In physiological pH, HA is negatively charged [68], and a possible mechanism would be that negatively charged bacteria are repelled by the negatively charged HA surface. The mechanism of the antibacterial effect of the nHA used in this study may depend on several factors and needs further investigation.

## 5. Conclusions

The coating used in this study showed nanosized HA crystals homogeneously spread over a Ti substrate in a 20–40 nm thick layer, which was superhydrophilic. The nHA layer showed a significant antibacterial effect, varying from 33% to 44.5% reduction, for 2 and 24 h biofilms, respectively, using *S. epidermidis* and *P. aeruginosa*, and was similar for both the Gram-positive and Gram-negative strains. The antibacterial effect seemed to be physicochemical rather than biological, which reduces the risk of bacterial resistance. This can be of benefit on implants in a clinical setting. Future research may investigate the antibacterial mechanism responsible for the results of this study. In addition, an in vivo study can be performed to investigate the antibacterial effects in an animal model.

## Figures and Tables

**Table 1:** EDX analysis of Ti and nHA-coated Ti of grades 2 and 4 (atomic percent).

Element	C	O	Ti	Ca	P
Ti grade 4	3.13 ± 0.23	12.90 ± 0.45	83.97 ± 0.61	-	-
nHA, Ti grade 4	2.06 ± 0.25	28.09 ± 1.09	69.11 ± 1.58	0.42 ± 0.15	0.32 ± 0.13
Ti grade 2	2.65 ± 0.13	25.61 ± 1.41	71.74 ± 1.46	-	-
nHA, Ti grade 2	1.75 ± 0.19	30.09 ± 1.25	67.72 ± 1.28	0.25 ± 0.04	0.19 ± 0.05

**Table 2:** Surface roughness values of Ti and nHA-coated Ti of grades 2 and 4.

Substrate	Sa (µm)	Sdr (%)	Sds (1/µm^2^)
Ti grade 4	0.23 ± 0.04	6.20 ± 1.96	0.20 ± 0.02
nHA, Ti grade 4	0.21 ± 0.05	5.29 ± 1.06	0.26 ± 0.03
Ti grade 2	0.25 ± 0.04	7.51 ± 1.14	0.22 ± 0.01
nHA, Ti grade 2	0.27 ± 0.05	7.10 ± 0.38	0.24 ± 0.03

## Data Availability

The original contributions presented in this study are included in this article; further inquiries can be directed to the corresponding author. The data are available on request from the authors.
